# Recipient-specific antibodies in HSCT: current knowledge and future perspectives

**DOI:** 10.3389/fimmu.2025.1621252

**Published:** 2025-07-03

**Authors:** Annamaria Pasi, Carmen Tania Prezioso, Patrizia Comoli, Ilaria Sbarsi, Rosalia Cacciatore, Giovanna Giorgiani, Santina Recupero, Paola Bergamaschi, Margherita Torchio, Alessia Taurino, Giulia Losi, Caterina Zerbi, Antonio Bianchessi, Irene Defrancesco, Nicola Polverelli, Marco Zecca, Cesare Giuseppe Perotti

**Affiliations:** ^1^ Immunohematology and Transfusion Service, Fondazione IRCCS Policlinico San Matteo, Pavia, Italy; ^2^ Pediatric Hematology and Oncology, Fondazione IRCCS Policlinico San Matteo, Pavia, Italy; ^3^ Cell Factory and Pediatric Hematology/Oncology Unit, Department of Mother and Child Health, Fondazione IRCCS Policlinico San Matteo, Pavia, Italy; ^4^ Division of Hematology, Fondazione IRCCS Policlinico San Matteo, Pavia, Italy

**Keywords:** hematopoietic stem cell transplantation, recipient-specific HLA antibodies, donor-specific anti-HLA antibodies, alloantigen-induced immune responses, complement-mediated endothelial injury, haploidentical stem cell transplantation, transplant immunology

## 1 Introduction

Allogeneic hematopoietic stem cell transplantation (HSCT) has significantly advanced the treatment of numerous hematological disorders. Advances in haploidentical transplantation have broadened access to this life-saving therapy, even for patients lacking fully matched donors (1–4). In this context, the role of donor-specific anti-HLA antibodies (DSA) in graft failure and delayed engraftment is well established, to the extent that pre-transplant screening for DSA has become standard practice in many centers (5–7). Conversely, considerably less attention has been devoted to another humoral immune factor: recipient-specific antibodies (RSA). Screening for DSA prior to transplantation—particularly those capable of complement fixation—is now standard practice and often guides interventions such as plasma exchange, administration of rituximab, and intensified immunosuppressive therapy in patients deemed at high immunological risk (8, 9). These practices underscore the clinical relevance of antibody-mediated complications in HSCT and offer a conceptual framework for evaluating the potential impact of RSA as well.

## 2 Clinical impact of Recipient-Specific Antibodies (RSAs)

### 2.1 Mechanisms of RSA-mediated damage

Recipient-specific antibodies (RSA) are antibodies present in the donor that recognize the recipient’s HLA antigens. Their development is often associated with previous allo-sensitization events, such as pregnancy in multiparous female donors, blood transfusions, or previous transplants (10). Once transferred during HSCT, RSAs can bind to recipient tissues, activate complement, and contribute to endothelial injury and inflammatory responses. Mechanistically, RSAs could act similarly to DSAs by triggering antibody-dependent cellular cytotoxicity (ADCC) or complement-dependent cytotoxicity (CDC), resulting in endothelial cell activation, loss of vascular integrity, and the creation of a pro-inflammatory microenvironment (11–13).

### 2.2 Clinical evidence and potential implications

Although the clinical relevance of RSAs is not as well established as that of DSAs, emerging evidence suggests they may play a non-negligible role in immune modulation after transplantation. Delbos et al. (14) reported an increased incidence of acute and chronic GVHD in recipients of transplants from donors harboring class II anti-HLA antibodies. Sadowska-Klasa et al. (15) hypothesized that RSAs may mediate endothelial activation via complement pathways, contributing to complications such as veno-occlusive disease (VOD) and transplant-associated thrombotic microangiopathy (TA-TMA). Post-transplant complications, such as engraftment syndrome (ES), cytokine release syndrome (CRS) in haploidentical transplantation with cyclophosphamide-based GVHD prophylaxis, cardiotoxicity, TA-TMA, and veno-occlusive disease/sinusoidal obstruction syndrome (VOD/SOS), share a common pathogenic mechanism centered on endothelial injury. This injury originates from a subclinical baseline condition, which is exacerbated by pro-inflammatory and pro-thrombotic events, including cytokine release (e.g., TNF-α, IL-6), complement cascade activation, reduced nitric oxide (NO) bioavailability, and elevated levels of angiopoietin-2, von Willebrand factor (vWF), and high mobility group box 1, potentially resulting in multiorgan failure (16–18). To date, TA-TMA remains the only syndrome with a clearly demonstrated association with recipient-specific antibodies (RSA) (15), as RSA may activate complement and directly damage the endothelium. Although direct evidence linking RSA to other endothelial complications is currently lacking, their shared endothelial pathophysiology supports the hypothesis that RSA could similarly contribute to these syndromes, warranting further targeted research. Additionally, Ciurea et al. described a haploidentical transplantation case in which RSA transfer was associated with early endothelial injury and adverse outcomes (19).

This relative omission in clinical practice may stem from various factors: the perception of low RSA levels, the lack of routine testing on donor samples, or the hypothesis that their impact might be less significant compared to that of DSAs. Recent reviews (20) have mainly emphasized the need to start considering the potential clinical role of RSAs and to investigate possible management parallels with DSAs, as current evidence is still too limited to draw definitive conclusions.

### 2.3 Immunologic modulation and RSA pathogenicity

One possibility is that RSAs contribute to the creation of a pro-inflammatory environment in the period immediately following transplantation, amplifying tissue damage triggered by conditioning regimens or subclinical allogeneic reactivity. In particular, RSAs capable of binding complement may have greater pathogenic potential, suggesting the use of functional assays, such as the C1q binding test, to identify clinically relevant cases. RSAs could thus act more as immunological “modulators” rather than direct barriers to engraftment, influencing the threshold for the development of GVHD, endothelial dysfunction, or chronic graft failure.

### 2.4 NIMA tolerance and maternal alloimmunization: a dual immunological legacy in haploidentical transplantation

In haploidentical transplantation, the mismatched donor haplotypes are referred to as non-inherited maternal antigens (NIMA) or non-inherited paternal antigens (NIPA). Due to fetal exposure to maternal HLA antigens during pregnancy, which may induce partial immunological tolerance, grafts from NIMA-mismatched donors are generally considered less immunogenic than those from NIPA-mismatched donors. Accordingly, several studies have demonstrated that NIMA-mismatched haplo-HSCT is associated with a significantly lower incidence of acute graft-versus-host disease (aGVHD) compared to NIPA-mismatched transplants. Although this evidence supports a tolerogenic effect induced during gestation, it is important to note that a substantial proportion of pregnant women develop HLA antibodies against paternal antigens. The mother encounters inherited paternal antigens (IPA) during adulthood, when her immune system is fully mature and immunocompetent. During pregnancy, she has approximately a 50% probability of mounting both humoral and cellular immune responses against the IPA haplotype.

**Figure 1:**
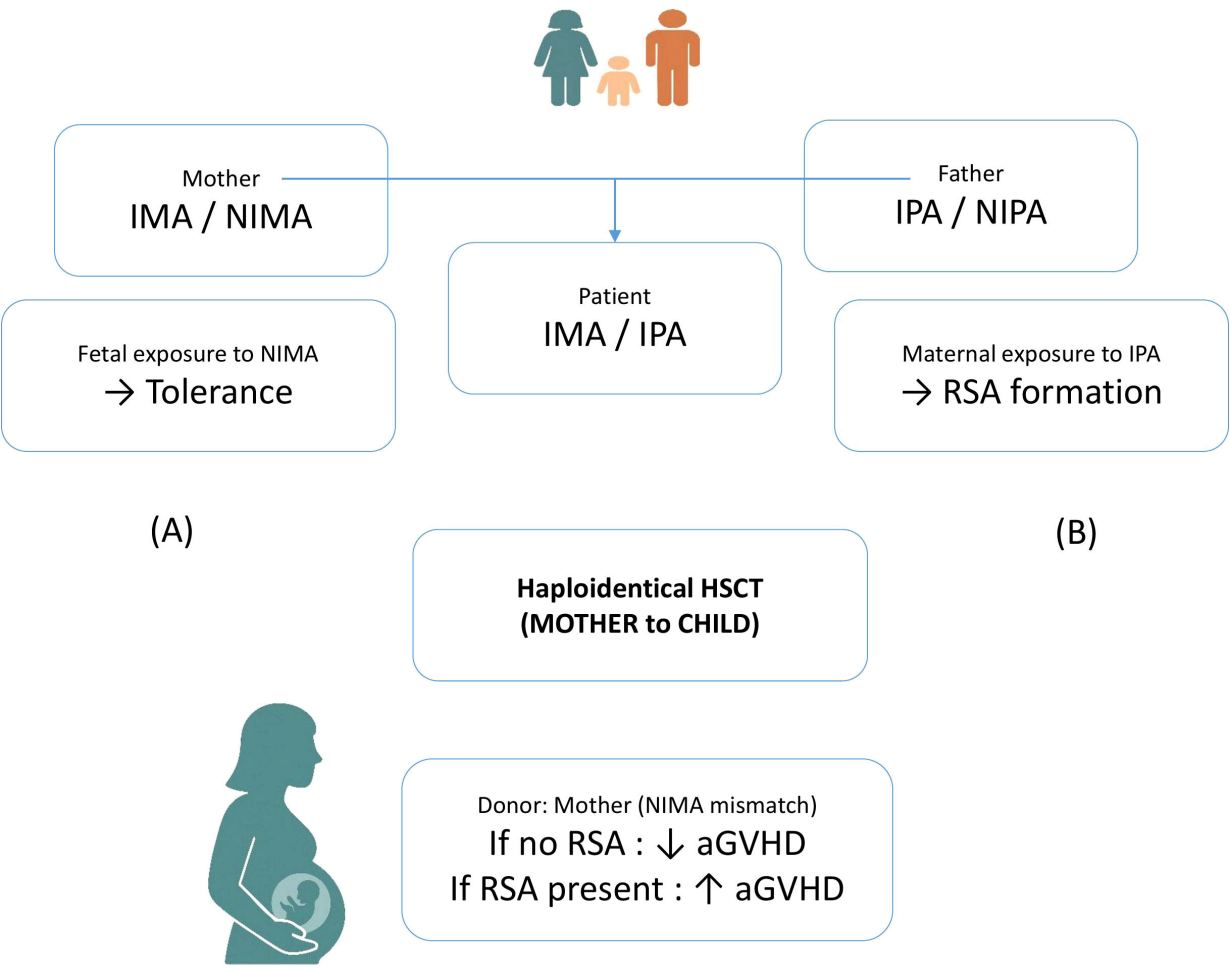
Schematic representation of dual immunological mechanisms occurring during pregnancy. **(A)** The fetus is exposed to non-inherited maternal antigens (NIMA), which may induce immune tolerance. **(B)** Conversely, the mother develops B e T cell immunity becoming sensitized to inherited paternal antigens (IPA) expressed by the fetus, this potentially leads to the formation of recipient-specific antibodies (RSAs).

In this context, the development of recipient-specific antibodies (RSAs) in multiparous mothers against the child’s IPA haplotype may adversely affect transplant outcomes, potentially negating the immunological advantage often attributed to maternal donors (21–23) (**Figure 1**). Future studies could be useful to clarify the interplay between NIMA-induced tolerance and maternal RSA formation against paternal antigens, and how these mechanisms impact donor selection and post-transplant outcomes (**Figure 1**).

### 2.5 Gender and reproductive history as risk factors

Gender-related and reproductive history–related immunologic sensitization is therefore a critical area that warrants further investigation. Should the clinical relevance of RSAs be confirmed, integrating targeted clinical strategies to mitigate the effects of prior sensitization could prove useful and might lead to modifications in current donor screening protocols and risk management approaches.

### 2.6 Technological advances in RSA detection

Defining clinically significant thresholds for RSAs would be crucial to standardizing diagnostic and therapeutic protocols at an international level, potentially promoting greater uniformity in the management of patients undergoing HSCT from haploidentical or partially matched donors (24).

Luminex technology has represented a significant methodological advance, enabling precise identification and quantification of RSAs thanks to its high sensitivity and specificity (25, 26). It has greatly facilitated the investigation of correlations between the presence and intensity of RSAs (measured by MFI) and post-transplant clinical outcomes. Although no validated thresholds currently exist for RSA interpretation, mean fluorescence intensity (MFI) values commonly used for DSA, typically >1,000 to indicate low-level sensitization and >5,000 for antibodies with clinical relevance, could serve as a preliminary reference. These values are supported by EBMT consensus guidelines (6). Aligning RSA interpretation with established DSA criteria may support more consistent risk assessment and guide future standardization efforts. Complement-binding functional assays, such as the C1q binding test, provide additional valuable information on the pathogenic potential of these antibodies.

### 2.7 Future perspectives on RSA screening and management

From a clinical perspective, the selective integration of RSA screening could represent a rational strategy. It could be especially considered for donors with a history of multiple pregnancies, and possibly for those with prior transfusion events, in whom the identification of significant RSAs might guide targeted therapeutic choices or influence donor selection.

However, the lack of large prospective studies makes it difficult to draw definitive conclusions about the clinical need for RSA screening. Prospective multicenter studies with harmonized methodologies and functional characterization of RSAs would be useful to assess whether integrating RSA screening into clinical practice is appropriate. In parallel, the development of therapeutic strategies to mitigate the effects of pathogenic RSAs—such as plasmapheresis, immunoadsorption, or complement inhibition—could offer new therapeutic options.

## 3 Discussion

In conclusion, recipient-specific antibodies represent a fascinating yet still underexplored aspect of transplant immunology. Preliminary evidence suggests that they may contribute to shaping the immune environment after HSCT, influencing the risk of GVHD, endothelial injury, and long-term transplant success. In contrast to donor-specific antibodies (DSAs), which are more clearly associated with graft rejection and engraftment failure, RSAs may play a distinct pathogenic role, particularly in the context of GVHD and immune modulation. Recognizing these differences could help to refine risk stratification and to outline new strategies for donor evaluation. RSAs should be considered as a potential piece of the complex mosaic of immune reactivity in HSCT. As research in this field progresses, integrating RSAs into a broader vision of transplant immunology could, in our opinion, broaden horizons for improving clinical outcomes.
